# Supplementary figures and images for: Germination characteristics among different sheepgrass (Leymus chinensis) germplasm during the seed development and after-ripening stages
Source: PeerJ. 2019 Apr 10;7:e6688. doi: 10.7717/peerj.6688 (PMC6462180; doi:10.7717/peerj.6688)

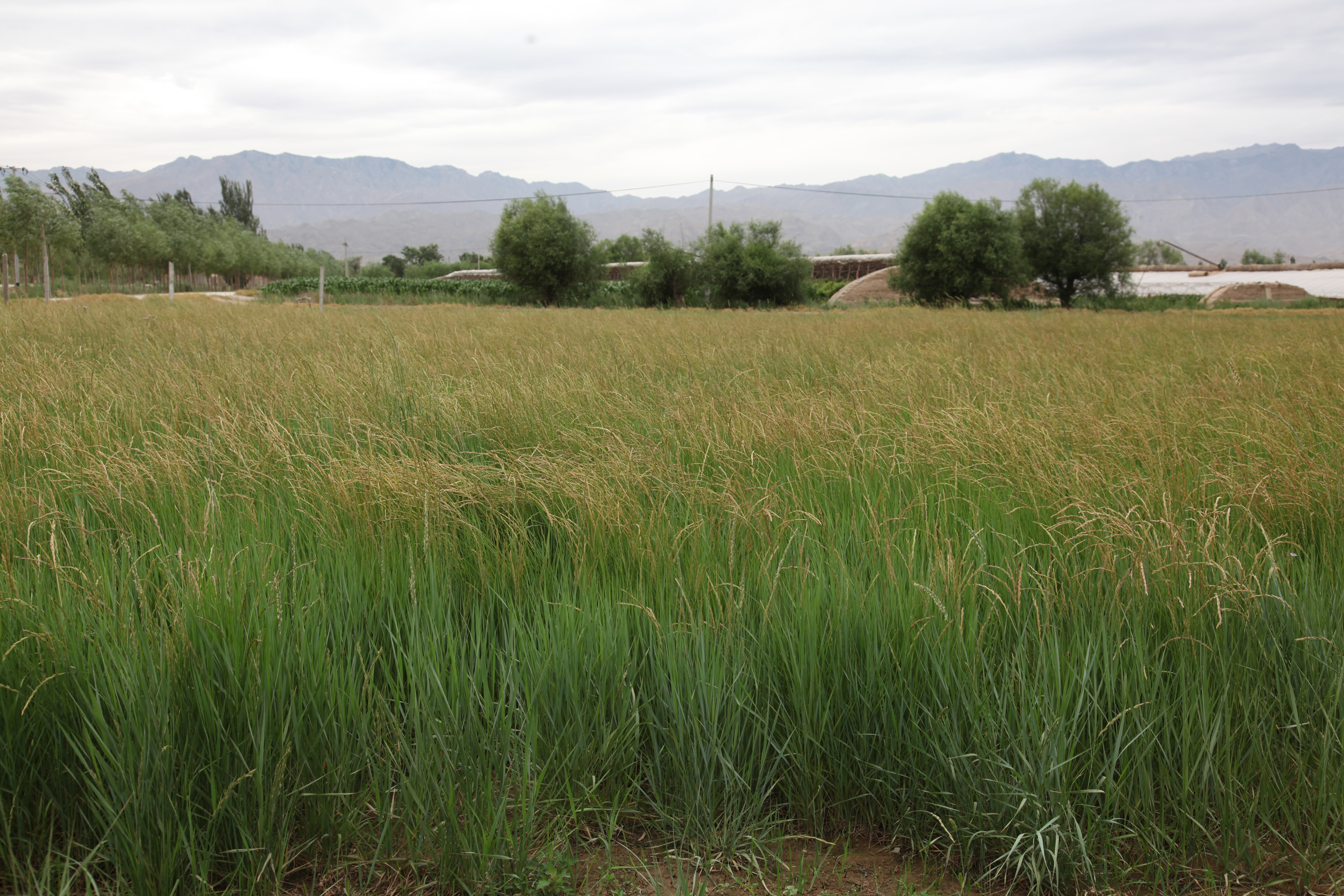

Supplement: Supplemental Information 2 — Population photo of sheepgrass at seed maturity period. Photograph by Gongshe Liu, Xiaoxia Li. [file peerj-07-6688-s002.jpg]
